# Unique Antibody Responses to Malondialdehyde-Acetaldehyde (MAA)-Protein Adducts Predict Coronary Artery Disease

**DOI:** 10.1371/journal.pone.0107440

**Published:** 2014-09-11

**Authors:** Daniel R. Anderson, Michael J. Duryee, Scott W. Shurmur, John Y. Um, Walter D. Bussey, Carlos D. Hunter, Robert P. Garvin, Harlan R. Sayles, Ted R. Mikuls, Lynell W. Klassen, Geoffrey M. Thiele

**Affiliations:** 1 Department of Internal Medicine, Division of Cardiology, University of Nebraska Medical Center, Omaha, NE, United States of America; 2 Veterans Affairs Nebraska-Western Iowa Health Care System, Research Service, Omaha, NE, United States of America; 3 Department of Internal Medicine, Division of Rheumatology, University of Nebraska Medical Center, Omaha, NE, United States of America; 4 Department of Surgery, Division of Cardiothoracic Surgery, University of Nebraska Medical Center, Omaha, NE, United States of America; 5 Department of Pathology and Microbiology, University of Nebraska Medical Center, Omaha, NE, United States of America; Indiana University School of Medicine, United States of America

## Abstract

Malondialdehyde-acetaldehyde adducts (MAA) have been implicated in atherosclerosis. The purpose of this study was to investigate the role of MAA in atherosclerotic disease. Serum samples from controls (n = 82) and patients with; non-obstructive coronary artery disease (CAD), (n = 40), acute myocardial infarction (AMI) (n = 42), or coronary artery bypass graft (CABG) surgery due to obstructive multi-vessel CAD (n = 72), were collected and tested for antibody isotypes to MAA-modifed human serum albumin (MAA-HSA). CAD patients had elevated relative levels of IgG and IgA anti-MAA, compared to control patients (p<0.001). AMI patients had a significantly increased relative levels of circulating IgG anti-MAA-HSA antibodies as compared to stable angina (p<0.03) or CABG patients (p<0.003). CABG patients had significantly increased relative levels of circulating IgA anti-MAA-HSA antibodies as compared to non-obstructive CAD (p<0.001) and AMI patients (p<0.001). Additionally, MAA-modified proteins were detected in the tissue of human AMI lesions. In conclusion, the IgM, IgG and IgA anti-MAA-HSA antibody isotypes are differentially and significantly associated with non-obstructive CAD, AMI, or obstructive multi-vessel CAD and may serve as biomarkers of atherosclerotic disease.

## Background

Inflammation is thought to be central in the pathogenesis of atherosclerosis [1], [2] and acute myocardial infarction (AMI) [3]. Additionally the reduction of inflammatory biomarkers has been shown to be of clear cardiovascular benefit [4]. However, the driving mechanism(s) of cardiovascular inflammation is/are uncertain. Modification of proteins, such as lipoproteins and the formation of protein-adducts, is one mechanism that has been associated with the development and/or progression of atherosclerotic disease [5]–[9]. These modified proteins have been found in the circulation [10], [11] and in atherosclerotic lesions of patients with atherosclerotic disease [5], [8], [12]–[14]. However, the exact direct and/or indirect mechanism(s) by which modified proteins result in cellular dysfunction, [14] immune sensitization, [15]–[19] tissue inflammation, and atherosclerotic plaque formation and rupture is not fully known.

Malondialdehyde (MDA), with the organic compound formula CH_2_(CHO)_2_, is generated as a result of oxidative degradation of lipids with formation of lipid peroxides, a process known as lipid peroxidation [9]. MDA is a mediator or marker of inflammation that has been associated with atherosclerosis and cardiovascular disease (CVD) [5], [8], [20]–[24]. More recently, it has been demonstrated that MDA can break down to form acetaldehyde (AA), [9] and research has shown that AA in the presence of MDA forms a unique malondialdehyde**–**acetaldehyde (MAA) adduct [25]. This MAA-adduct structure is a dihydropyridine (4-methyl-1,4-dihydropyridine-3,5-dicarbaldehyde) which predominately modifies the epsilon-amine of lysine, is highly stable, is the immunodominant MDA-epitope, and biologically functions as a potent immunoenhancing factor [5], [26]–[28]. Importantly, MAA-adducted macromolecules have been shown to be cytotoxic, proinflammatory and result in a robust specific adaptive immune response to the MAA structure, the MAA-adducted macromolecule, and/or the hapten-carrier structure of the MAA-adducted macromolecule [5], [26], [27], [29].

Previous studies by our group showed the presence of MAA-modified proteins in aortic tissue of rabbits on a high fat diet [8] and aortic tissue of JCR diabetic/atherosclerotic rats [5]. Others have also shown the association of serum anti-MAA antibodies with diabetes [30], [31], and serum MAA-immune complexes with cardiovascular events in type 2 diabetic patients [32]. These data strongly suggest MAA has a role in CVD.

In this report, we specifically determined in humans the presence of MAA-adducted macromolecules in atherosclerotic plaques and evaluate the antibody isotype response to MAA (i.e. IgM, IgG, IgA) as it relates to cardiovascular disease and cardiovascular events.

## Methods

### Patients and Sample Collections: The Nebraska Cardiovascular BioBank and Registry

Research which included the optional collection and banking of biological samples protocols were approved by the Institutional Review Board (IRB) of the University of Nebraska Medical Center under strict ethical guidelines. All studies performed on patient samples conformed to the declaration of Helsinki. Informed written consent for the collection and use these tissues was obtained from each patient prior to donation when patients underwent elective procedures. With AMI patients, the IRB approved an initial waiver of consent for the collection of the tissue as to not delay treatment (i.e. door-to-balloon times). However, informed written consent was obtained from AMI patients after recovery and before hospital discharge. Steps during collection of these excess tissues were designed and monitored to ensure no delay in treatment occurred. Regarding sharing of data elements, our research subjects were not consented for public sharing of individual data elements, thus, we are restricted in presenting these individual data elements in a public database.

Over a six-month period, tissue and serum samples were collected from: 1) Patients undergoing cardiac catheterization for the evaluation of chest pain or cardiac ischemia but did not have an acute myocardial infarction (AMI) or non-ST elevated MI (NSTEMI), 2) Patients who presented with an acute myocardial infarction (AMI) and underwent emergent cardiac catheterization and intervention; 3) Patients undergoing elective coronary artery bypass graft surgery (CABG); and, 4) Volunteer controls (Controls).

Patient and Control demographics are presented in Table 1. In brief, blood and tissue was collected from 82 Controls which was a convenience sample of patients who reported well to excellent health and no diagnoses or treatment of atherosclerotic disease, hypertension, hyperlipidemia or diabetes. Coronary artery disease (CAD) patients included 40 patients who presented with chest pain without AMI or NSEMI who had CAD which was less than 50% lesion area on diagnostic catheterization and did not require intervention (Non-Obstructive CAD), 42 patients who presented with ST-elevated AMI (Acute MI) and underwent emergent cardiac catheterization and intervention with collection of blood at the time of intervention, and 72 patients who on diagnostic catheterization had significant multi-vessel obstructive CAD without AMI or NSTEMI and presented for elective CABG (Multi-Vessel Obstructive CAD). AMI patients presented to the Emergency Department with a history of 385 average minutes of chest pain prior to presentation. Our CABG group of patients underwent one (0%), two (17%), three (37%), four (40%) or five (6%) vessel bypass grafting.

**Table 1:** Patient demographics.

	Controls	Non-ObstructiveCAD	Acute MyocardialInfarction	ObstructiveMulti-vessel CAD
**Number**	82	40	42	72
**Age**	50.4±12.1	56.6±10.2^a^	59.2±10.3^a^	64.0±10.3^ab^
**Male**	18	23	27	55
**Female**	64	17	15	17
**hsCRP**	3.7±6.3	6.3±9.7	6.3±10.4	11.0±15.4^c^
**IL-6**	1.1±4.1	3.9±7.9^d^	22.2±43.1^de^	6.9±13.6^d^
**Total Cholesterol**	183.3±42.9	180.5±60.6	177.1±55.3	155.3±41.7^f^
**LDL Cholesterol**	103.2±31.9	93.4±44.7	101.2±40.7	82.9±33.6^g^
**HDL Cholesterol**	51.8±14.7	41.7±14.9	35.3±9.4	37.4±11.9^g^
**History of Tobacco**	40%	65%	60%	67%
**History of Alcohol**	*#	45%	33%	35%
**Hypertension**	*#	93%	86%	86%
**Diabetes**	*#	43%	52%	46%
**Ace Inhibitor**	*#	33%	34%	43%
**Statin**	*#	40%	40%	88%

a P<0.01 significantly increased compared to control.

b P<0.05 significantly increase compared to Non-Obstructive and Acute AMI.

c P<0.001 significantly increased compared to control.

d P<0.01 significantly increased compared to control.

e P<0.001 significantly increased compared to control, Non-Obstructive CAD, and Obstructive Multi-Vessel CAD.

f P = 0.03 significantly decreased compared to control, Non-Obstructive CAD, and acute.

g P<0.02 significantly decreased compared to control, acute.

*#Control subjects reported they were healthy with no medical problems or medications.

The culprit lesion in AMI patients was identified using routine coronary angiography. In clinically indicated cases, thrombus aspiration of the AMI culprit vessel occurred using a Medtronic Export XT Aspiration Catheter. This “aspirated sample” was given to research staff for processing. This aspirate was placed in a 0.2 micron filter basket and washed with non-heparinized normal saline. In 30–35% of all aspirates, the tissue collected was 1–5 mm in size. The sample was either transferred to a vial containing 1 mL of RNA later (Qiagen, Valencia, CA) and archived at −80°C, or fixed in formaldehyde for histology. With all catheterization patients including AMI, blood was collected for serum and plasma upon insertion of the venous sheaths. These samples were immediately centrifuged in separator tubes and archived at −80°C. During CABG surgery, and prior to heparin administration or being placed on a bypass pump, serum and plasma was collected and immediately processes and archived.

### Patient Demographics testing

Data found in Table 1 for age, sex, total cholesterol, LDL, HDL, tobacco, alcohol, hypertension, diabetes, ace inhibitor, and statin use were extracted from the patient records in accordance with UNMC IRB approval. High sensitivity CRP was determined using a BNII Nephelometer (Siemens, Munich, Germany), and the data expressed in mg/L. IL-6 was determined by Enzyme linked immunosorbent assay (ELISA) using the OptEIA ELISA kit (Pharmingen, Palo Alto, CA) according to manufacturer’s instructions.

### Determination of Circulating Antibodies to MAA

Serum from all patients was screened for the presence of the immunoglobulin (Ig)-M, IgG, and IgA isotypes of anti-MAA antibodies. Briefly, for these experiments aqueous human albumin (Alb) (Talecris Biotherapeutics, Inc., Research Triangle Park, NC) was modified with malondialdehyde and acetaldehyde (2∶1 molar ratio) as previously reported [5], [25]. This 2∶1 ratio of malondialdehyde and acetaldehyde results in protein adduct that is predominantly MAA [5]. Enzyme-linked immunosorbent assay (ELISA) plates were coated with MAA-Alb or Alb, and human IgM, IgG or IgA isotype standards to be used for extrapolation of relative antibody concentrations (Sigma Chemical Company, St. Louis, MO). Plates were incubated overnight at 4°C, washed, blocked with 2% bovine serum albumin, and incubated with patient serum at a 1∶1000 dilution. Following incubation at 37°C for 1 hour, a secondary HRP goat anti-human antibody specific for IgM (Fc5u fragment specific), IgG (Fcγ specific) or IgA (α chain specific) (Jackson ImmunoResearch, West Grove, PA) was added. Plates were developed using TMB substrate, and after 30 minutes absorbance determined at 450 nm using an MRXII microplate reader (Dyantech, Chantilly, VA). Relative concentrations of anti-MAA antibody were extrapolated from the isotype standard curve. Data is presented as relative mg/L of the specific anti-MAA antibody isotype detected in the assay.

For total serum immunoglobulin determination, serum from AMI patients collected at the time of AMI and 24 hours post-AMI were analyzed using a BNII Nephelometer (Siemens, Munich, Germany), and the data expressed in g/L.

### Determination of Circulating Antibodies to MDA-LDL and MAA-LDL

Serum from all patients was screened for the presence of anti-MDA-LDL and MAA-LDL IgG antibodies. Human LDL (Biomedical Technologies, Inc, Ward Hill, MA) was modified with MDA as previously described [33]. Briefly, human LDL was reacted with 0.2 M MDA for 3 hours at 37 degrees. Human LDL was MAA modified by reacting 2 mM MDA with 1 mM acetaldehyde as previously described [25]. ELISA plates were coated with MDA-LDL, MAA-LDL, and LDL alone and serum tested for reactivity as describe above.

### Culprit AMI Occlusion: Tissue Histology and MAA Identification

Tissue samples from the aspirated culprit occlusion of AMI patients were collected and placed in RNAlater for storage purposes. Preserved samples were paraffin-embedded, sectioned and stained with Masson’s Trichrome reagent. Stained tissues were slide scanned using the iScan Coreo Au Slide Scanner (Ventana Medical Systems, Inc., Tucson, AZ) and analyzed using ImageViewer (Bioimagene, Cupertino, CA). Detection of MAA-modified proteins was done using immunohistochemical techniques with a well characterized affinity purified polyclonal antibody to MAA as previously described [34]. Briefly, sectioned tissues were blocked with 5% goat serum, washed and incubated with the purified rabbit anti-MAA polyclonal antibody. Detection of anti-MAA was done using a Cy3 goat anti-rabbit IgG secondary antibody (Jackson ImmunoResearch Laboratories, Inc, West Grove, PA). Slides were mounted using Fluoromount-G (Southernbiotech, Birmingham, AL) and flouroescence detected using a Zeiss 510 Meta Confocal Laser Scanning Microscope confocal microscopy (North Chesterfield, VA). Images were analyzed using LSM imaging Software (Zeiss).

### Statistical Analysis

Anti-MAA antibody isotype values (IgA, IgG, and IgM) were highly skewed; for this reason the natural logs of anti-MAA values were used in the analyses. Initial comparisons of mean anti-MAA antibody levels between groups using ANOVA were made using Student-Newman-Keuls post-hoc method analysis of differences between predicted means from ordinary least squares (OLS) regression models controlling for age, sex, tobacco history, diabetes, hypertension and statin use. Multinomial logistic regression models, both with only anti-MAA levels as predictors and then also controlling for age sex, tobacco history, diabetes, hypertension and statin use, were used to determine if the type of cardiovascular event (CABG or Acute MI versus stable) could be predicted from anti-MAA antibody levels. Logistic regression models similar to the multinomial models were used to distinguish between a combination of CABG and Acute MI (event) versus stable CAD (no event). Further logistic regression models were used to distinguish between CABG and Acute MI among those with an event. All analyses were conducted using STATA version 12 (StataCorp LP, College Station, TX).

## Results

Evaluation and comparison of 4 patient groups were undertaken and this study cohort is presented in the Table 1. These four groups included; 1) “Controls” – Patients without any history of CAD; 2) “Non-Obstructive CAD” - Patients that presented for cardiac catheterization with chest pain and CAD, but no evidence of an AMI; 3) “Acute MI” - Patients with CAD who presented with an AMI; and, 4) “Multi-Vessel Obstructive CAD” - Patients with severe CAD who presented for CABG surgery. Collections of all patient samples were independent and there was no overlap between study groups.

Review of the demographic table reveals our CAD patients (Non-Obstructive CAD, Acute MI, and Multi-Vessel Obstructive CAD) are well matched in regard to age, total cholesterol, LDL, HDL, hypertension and diabetes. However, Non-Obstructive CAD and Acute MI patients who were diagnosed with CAD at the time of catheterization, have a lower incidence of ACE inhibitor and statin use compared to patients with known CAD presenting for CABG surgery. These variations reflect the acute recognition of CAD in a portion of these patients and thus the implementation of cardiovascular risk factor management strategies. hs-CRP is elevated in all groups and significantly elevated in AMI and Multi-Vessel Obstructive CAD patients consistent with the literature [35]. IL-6 is significantly elevated in all groups compared to controls and significantly elevated in AMI patients which is consistent with the observation that this is an acute phase reactant [3]. Our Non-obstructive CAD, Acute MI and Multi-Vessel Obstructive CAD groups did have lower total cholesterol, HDL and LDL levels compared to the control group, which is consistent with appropriate lipid therapy and cholesterol reduction in our groups of patients with known CAD. Our control group did have elevated hs-CRP levels at 3.7±6.3 mg/ml and this level of inflammation would place our control group at an average relative vascular risk of a cardiovascular event versus the high risk of our cardiovascular event patients [36]. There was no association with anti-MAA antibody levels to IL-6, alcohol use, hsCRP or LDL (p>0.05). In regard to alcohol we were only able to determine a history of alcohol use and unable to quantitate the amount of alcohol consumption. There was no clinical record of alcohol induced cirrhosis or hepatitis and our lack of an association is consistent with prior reports wherein serum anti-MAA antibody levels do not increase with heavy alcohol use. Anti-MAA antibody levels only increased when alcoholic induced cirrhosis or hepatitis was clinically evident [37].

There was a greater proportion of females (n = 64) compared to males (n = 18) in our control group. However, there was no significant difference (p>0.1) in the relative concentrations of anti-MAA IgM (468.3±84, 281.5±45 mg/L), IgG (91.3±6.6 mg/L, 119.1±21 mg/L) or IgA (83.2±12 mg/L, 80.6±19 mg/L) (graph not shown) when comparing females to males, respectively.

**Figure 1:**
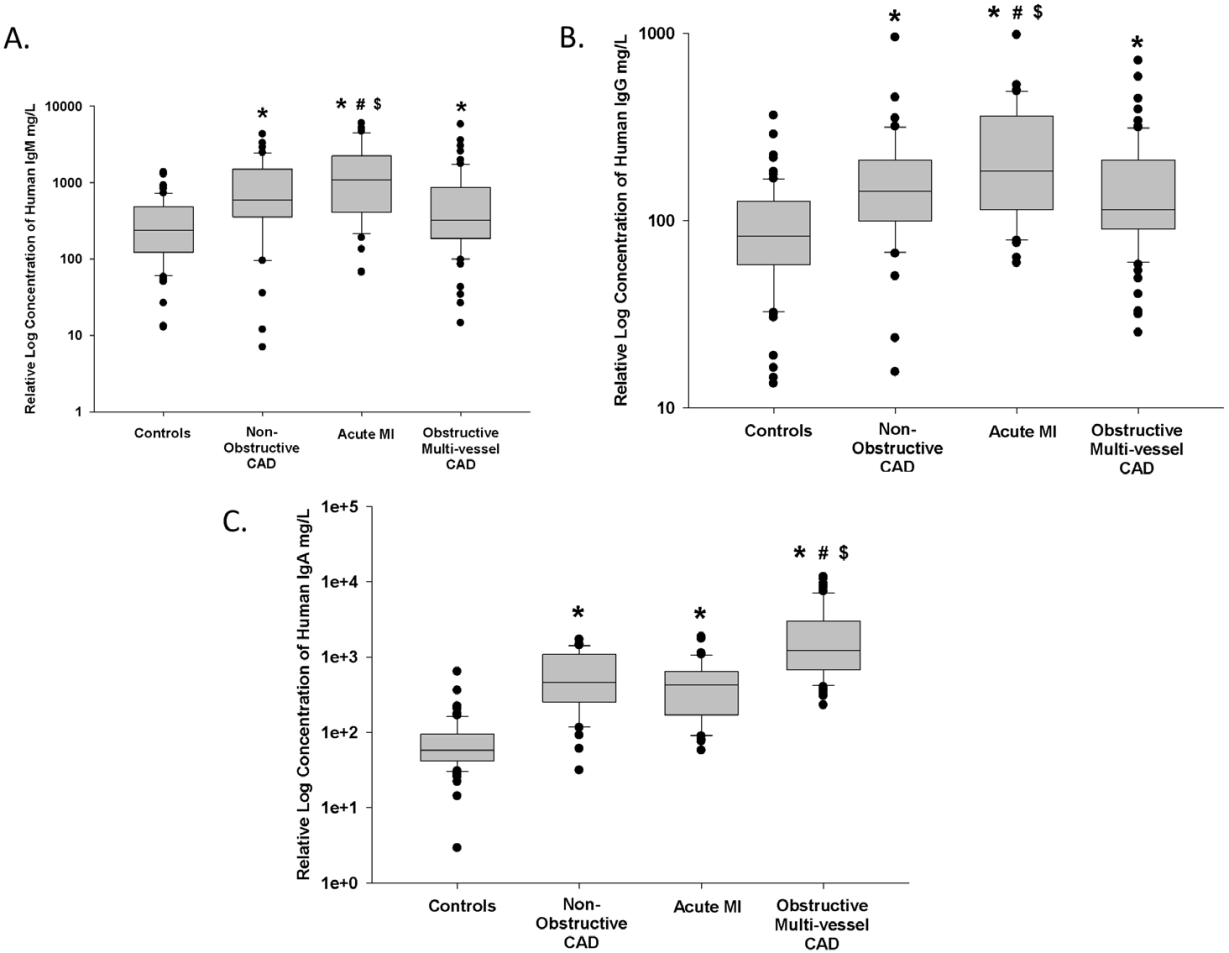
Relative Serum Concentrations of anti-MAA IgM, IgG and IgA Antibodies are Increased in Individuals with Coronary Artery Disease (CAD) and in Individuals who Present with an Acute Myocardial Infarction (AMI). CAD patients were grouped in the following categories; control patients (n = 82), patients with chest pain and CAD (Non-Obstructive CAD, n = 40), patients presenting with AMI (n = 42), and patients with significant Multi-Vessel Obstructive CAD requiring coronary bypass grafting (n = 72). Serum anti-MAA antibodies were evaluated for the isotypes IgM (Figure 1A), IgG (Figure 1B), and IgA (Figure 1C). *P<0.001 significantly increased compared to controls. #P<0.03 significantly increased compared to Non-Obstructive CAD. $P<0.003 significantly increased compared to Multi-Vessel Obstructive CAD.

Patients with acute MI had significantly higher levels of serum IgM anti-MAA (1603.9 mg/L) (p<0.01) compared to control patients (347.1 mg/L) (Figure 1A). The Multi-Vessel Obstructive group (700.0 mg/L) had increased levels over controls, but was not significantly elevated. In AMI samples, there was an increased IgM anti-MAA level compared to the Non-Obstructive group (1001.1 mg/L) (p = 0.026).

Patients with Non-Obstructive, Acute MI and Multi-Vessel Obstructive CAD had significantly higher relative levels of serum IgG anti-MAA (183.4 mg/L; 244.9 mg/L; 163.70 mg/L, respectively) (p<0.001) compared to control patients (97.4 mg/L) (Figure 1B). Acutely after AMI (within 60 minutes of chest pain), relative levels of anti-MAA IgG antibody (Figure 1B) were significantly elevated over Non-Obstructive (p = 0.027) or Multi-Vessel Obstructive CAD levels (p = 0.003).

**Figure 2:**
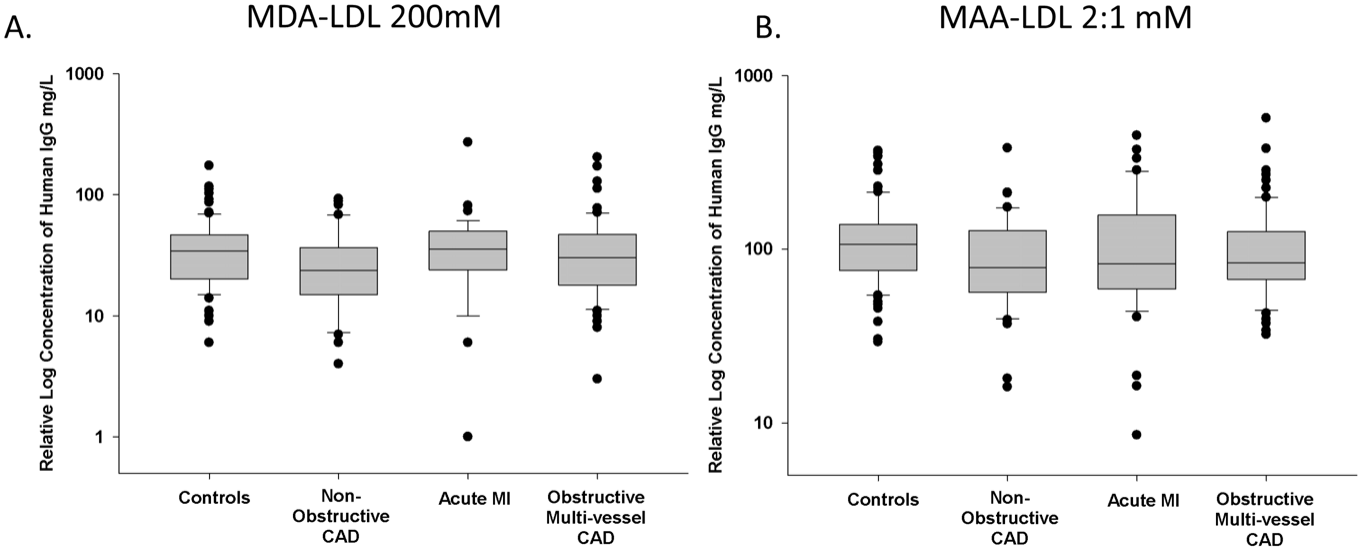
Relative Serum Concentrations of anti-MDA LDL and anti-MAA LDL IgG Antibody are not Different in Individuals with Coronary Artery Disease (CAD) and in Individuals who Present with an Acute Myocardial Infarction (AMI). CAD patients were grouped in the following categories; control patients (n = 82), patients with chest pain and CAD (Non-Obstructive CAD, n = 40), patients presenting with AMI (n = 42), and patients with significant Multi-Vessel Obstructive CAD requiring coronary bypass grafting (n = 72). Serum anti-MDA LDL (Figure 2A) and anti-MAA LDL (Figure 2B). There is no significant difference in serum antibody levels when comparing all study groups (p>0.5).

Patients with Non-Obstructive, Acute MI and Multi-Vessel Obstructive CAD had significantly higher relative levels of serum IgA anti-MAA (641.3 mg/L; 493.2 mg/L; 2423.9 mg/L, respectively) (p<0.001) compared to control patients (82.6 mg/L) (Figure 1C). The most dramatic changes were observed in the serum of the Multi-Vessel Obstructive group, in which a significant increase in IgA anti-MAA was observed (p<0.001) compared to both the acute MI and Non-Obstructive patient groups. Figure 2 demonstrates that here is no difference between our controls and study groups when we evaluated for serum antibody reactivity against MDA-LDL (Figure 2A) or MAA-LDL (Figure 2B).

In summary, anti-MAA antibody isotype evaluation reveals a pathogenic association of IgM (Figure 1A) and IgG (Figure 1B) isotypes with AMI, while serum IgA (not secretory IgA) is associated with chronic multi-vessel obstructive CAD (Figure 1C). These antibody levels are unique as compared to anti-MDA-LDL or anti-MAA-LDL antibody levels which did not relate to the presence or severity of CAD (Figure 2).

**Figure 3:**
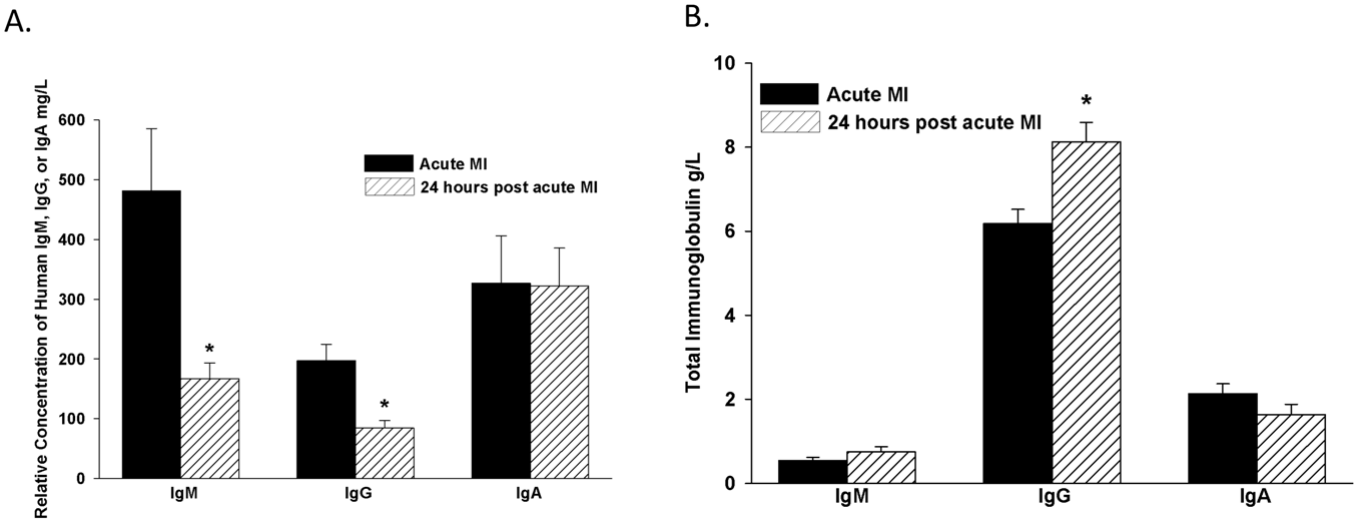
Serum Concentrations of IgM, IgG and IgA Antibodies 24 Hours post-AMI. A subgroup of patients (n = 10) were evaluated 24 hours post-AMI for the presence of circulating IgM, IgG and IgA anti-MAA antibody levels (Figure 3A) and the total serum IgM, IgG, and IgA concentrations (Figure 3B). Results are expressed as relative mg/L or g/L of Human IgM, IgG, and IgA using a standard curve. *P<0.01 significantly different comparing AMI and 24 hours post-AMI.

On a sub-group of AMI patients (n = 10) serum was collected 24 hours post-AMI and assayed for serum anti-MAA relative levels. 24 hours post-AMI, IgG anti-MAA antibody relative levels decreased 72.6% (p = 0.015), IgM anti-MAA decreased 71.6% (p<0.01), and IgA anti-MAA was unchanged (Figure 3A). To evaluate if this decrease in IgM and IgG was non-specific as a consequence of the AMI, total Ig concentrations were evaluated. Total IgM (0.54 g/L verses 0.75 g/L, p = 0.17), and IgA (2.13 g/L verses 1.63 g/L, p = 0.16) concentrations at the time of AMI compared to 24 hours post-AMI were not significantly different, respectively. Total IgG (6.18 g/L verses 8.13 g/L, p = 0.003) significantly increased at 24 hours post-AMI (Figure 3B) consistent with the reactive immune response associated with an AMI [3]. As detailed in the methods section, it is important to recognize that anti-MAA Ig levels presented in this manuscript are relative to a human isotype control and is not a true concentration value as is presented for total serum IgM, IgG and IgA. Thus, data in Figure 3A and 3B is presented to compare the relative change of anti-MAA relative levels 24 hours post-AMI in comparison to total serum Ig concentrations 24 hours post-AMI.

**Figure 4:**
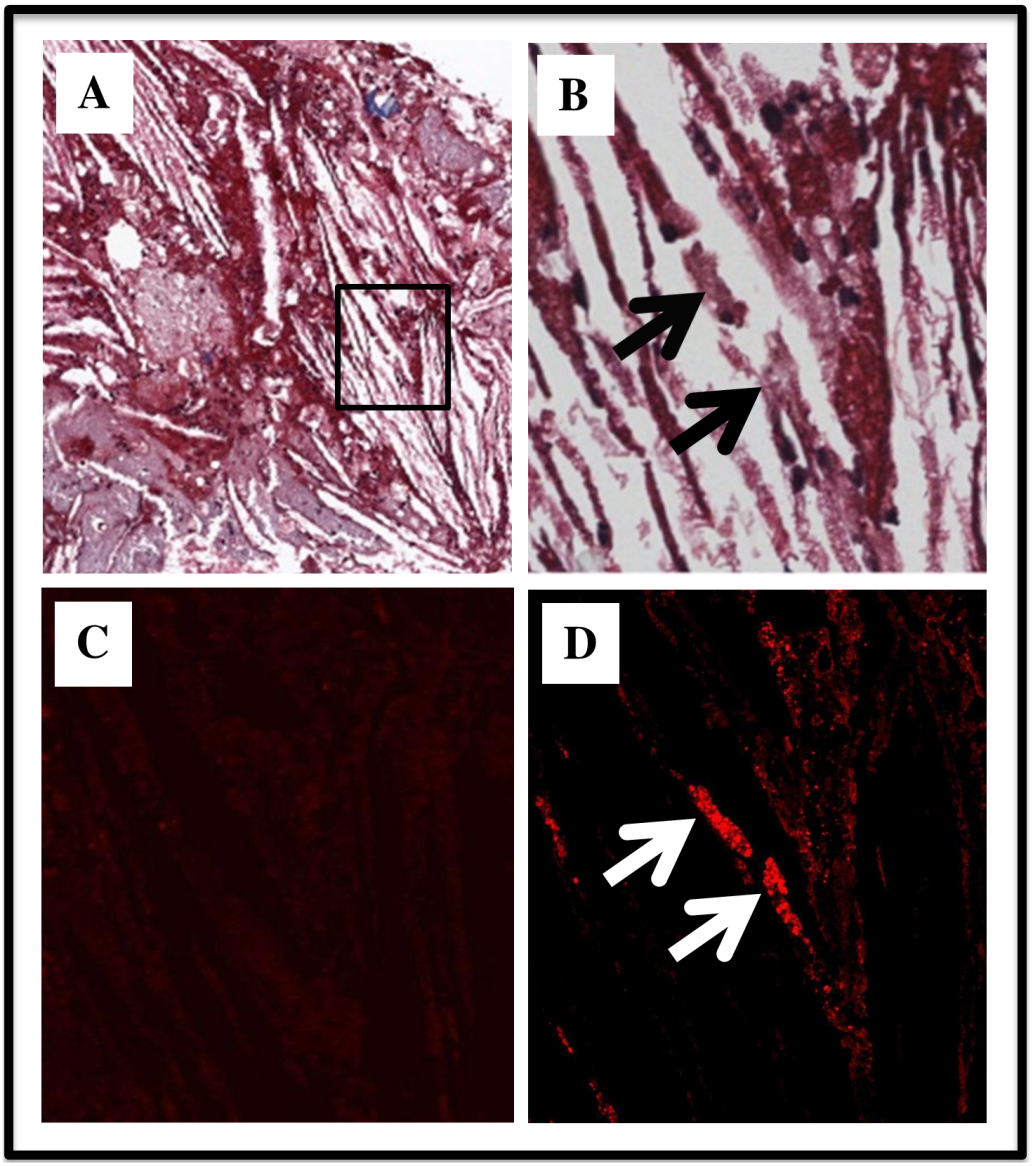
Light and Confocal Microscopy of MAA in the Culprit AMI Aspirated Tissue. Panel A and B illustrates a Masson’s Trichrome staining at low (20X) and high magnification (80X) with panel B as the inset box of panel A. Panel C is the rabbit IgG isotype control stain. Panel D illustrates the rabbit anti-MAA staining with Cy3 reporter (80X). Note the absence of collagen or fibrosis and the presence of cholesterol clefts in Panel A which are typical of an atheroma. Also note the localization of MAA in Panel D (white arrows) to cellular vacuolization and necrosis as noted by the arrows on the Masson’s Trichrome in Panel B (black arrows).

To evaluate the presence and possible pathogenic role of MAA-adducted proteins, cardiovascular tissue was evaluated for the presence of MAA-adducts. Confocal microscopy of the aspirated tissue from the culprit AMI lesion (Figure 4) illustrates cholesterol clefts typical of an atheroma, and demonstrates the presence of the MAA-adduct and localization of MAA to cellular debris. As illustrated by the sequential tissue sections, greater intensity of MAA-adduct staining (Figure 4D) is present in cells with more tissue vacuolization and loss of cellular histologic features (denoted by the arrows in Panel 4B).

## Discussion

In this report we investigated the role of MAA-adducts in the development and progression of atherosclerotic disease. We also investigated the utility of serum anti-MAA antibodies as biomarkers of atherosclerotic disease and clinical progression of coronary heart disease. The process by which proteins are MAA-adducted, as well as the implications of this adduction on cellular processes, immune sensitization, disease progression and cardiovascular events, are outlined (Table 2) and briefly discussed.

**Table 2:** MAA-Adduction and its Implications.

Mechanism of MAA-Protein Adduction
•Nucleophilic substitution (S_N_-2) reactions between Malondialdehyde (MDA), Acetaldehyde (AA) and the epsilon-amine of a lysine amino acid.
• MDA is a product of oxidative degradation of lipids with formation of lipid peroxides (i.e. lipid peroxidation). • AA as a product of MDA breakdown, alcohol metabolism, and present in tobacco smoke in high concentrations.
Characteristics of the MAA-Adduct
•MAA is the dominant product of MDA•Is a highly stable adduct•Highly immunogenic and serves as a potent adjuvant alone
Cellular impact of MAA-Adduction
•MAA is cytotoxic
• Induces apoptosis, autophagy and necrosis
•MAA up-regulates mRNA and Protein Expression of Pro-inflammatory Mediators
• Interleukin-6 • Tumor necrosis factor-alpha • Macrophage chemotactic protein-1
•MAA-adduction is a Potent Immune-Enhancing Factor
• Results in a robust adaptive immune response (class switching from IgM to IgG) to the
♦ MAA structure ♦ MAA-adducted macromolecule ♦ Hapten-carrier of the MAA-adducted macromolecule ♦ Co-adducted molecules (i.e. carbamyl-epitopes or other adducts)
Potential Cellular Impact of MAA-Adduction
•MAA may modulate protein function
• Modification of the regulatory lysine epsilon-amines
♦ Epsilon-amines are regulatory via acylation and de-acylation
• Modification of epsilon-amines of lysine is suspected to remove this regulatory element and result in protein dysfunction.

Modification of proteins such as lipoproteins and protein-adducts by lipid peroxidation or reactive oxygen species has been associated with the development and/or progression of atherosclerotic disease [5]–[8]. These modified proteins have been found in the circulation [10], [11] and in atherosclerotic lesions [12]–[14] of patients with atherosclerotic disease. Additionally, MDA- and 4-hydroxy-2-nonenal (4-HNE) modified lipoproteins have been shown to be immunogenic, generating autoantibodies against epitopes within the plasma apolipoprotein B-100 (ApoB 100) component of LDL (512 kDa) which have been variably associated with cardiovascular events [11], [12], [38]–[40]. The recent recognition that MAA is one of the terminal and stable adducts of MDA, [5] and that MAA-modified macromolecules initiate innate and acquired immune responses, [26], [27] has resulted in studies of MAA-adduction and/or its potential impact on cardiovascular disease and cardiovascular events.

MAA-adducted macromolecules result in robust and specific immune responses to the MAA structure, the MAA-modified protein, and/or the hapten-carrier structure of the MAA-modified protein [5], [26], [27]. The relationship of anti-MAA antibody isotype concentrations to the clinical presentation of CAD patients as presented in Figure 1 is consistent with literature in other inflammatory conditions [41]. Specifically, IgA anti-MAA antibodies appear to be associated with an inflammatory response which is associated with chronic stable progressive disease (i.e. stable CAD), while IgM and IgG isotypes appear to be associated with the pathologic inflammatory response associated active progressive disease (i.e. unstable CAD).

To date, numerous autoantigens and infectious agents have been reported to be involved in atherosclerotic disease, [42] including oxidized-LDL [43]. We have previously shown that in the atherosclerotic James C. Russell corpulent (JCR:LA-cp) rat serum antibodies react to oxidized-LDL and MAA-modified LDL, [5] and that MAA is a potent immunoenhancing factor [5], [26], [27]. Additionally, previous studies support the view that MAA-adducted proteins have the potential to be pathogenic, [29], [44] and that MAA-adducts elicit specific anti-MAA immune responses that are dependent upon scavenger receptors [45].

Elevated relative concentrations of anti-MAA antibody are associated with atherosclerotic disease and cardiovascular events as shown by the data presented in Figure 1. However we do not know the atheromatous burden and are not able to accurately define or delineate the presence of subclinical CAD in our “convenience” control group since they did not undergo a heart catheterization. But, these controls reported they were healthy and had no medical problems. Thus, although they may have had unrecognized or sub-clinical disease such as diabetes, they did not present with symptoms of occlusive atherosclerotic disease as compared to the other groups we studied. Despite these limitations, there was still a significant difference between these control patients and our patients with non-obstructive CAD and patients who had a cardiovascular event (i.e. AMI) or needed revascularization (i.e. CABG) due to obstructive CAD. This significant difference suggests that increases in relative anti-MAA antibody levels are associated with the symptoms of atherosclerotic disease, cardiovascular events and revascularization.

Figure 2 demonstrates there is no difference between our patient groups when using MDA-adducted LDL or MAA-adducted LDL as the antigen to determine anti-MDA-LDL or anti-MAA-LDL serum IgG antibody levels. This is consistent with data presented by Tsimikas and colleagues, which showed no significant association between serum levels of anti-MDA-LDL IgG with the extent and severity of CAD [22] or with the primary composite cardiovascular disease endpoint including ischemic stroke, myocardial infarction, new-onset unstable angina, acute coronary interventions, and vascular death during follow up [40].

MAA-modification of tissue proteins can have multiple biological effects. MAA-adduction, may result in perturbation of protein function by modifying the regulatory epsilon-amine group on lysine [46]. MAA-adduction may also be directly cytotoxic at the cellular level [29], [44], and may serve as an adjuvant in sensitization [5], [26], [27] to self-proteins. In this regard, MAA-adduction and immune sensitization may occur to multiple epitopes of the MAA-adducted protein including the MAA structure, the MAA-adducted cardiovascular protein and the hapten-carrier structure of the MAA-adducted protein [5], [26], [27]. Furthermore, if the MAA-adducted protein(s) is co-adducted or co-modified (i.e. citrulline [47], carbamyl-epitopes [48] or other adducts) it would be expected that a MAA-mediated humoral sensitization to these other adducts could also occur. For example, Kummu et al. [48] have shown the presence of cross-reactive serum antibodies in CAD patients that competitively bind carbamylated-LDL, MDA-LDL and MAA-LDL. Thus, the difference in antibody levels when using MAA-LDL or MAA-HSA as the capture antigen as presented in Figures 1 and 2, may reflect the binding of other serum LDL-autoantibodies which mask MAA-specific epitopes. MAA-adduction may be one mechanism for immune sensitization to LDL and/or co-adducted epitopes (multi-valiant antigens) that are associated with cardiovascular disease and cardiovascular events.

It is not clear how the antibody isotypes associate with or if these isotypes contribute to fibrous cap thinning, plaque rupture and AMI (i.e. IgG and AMI) or fibrous cap stabilization with chronic stable CAD (i.e. IgA and CABG patients) [43], [49]. These isotype differences between our study groups may reflect different immune phenotypes of atherosclerotic disease progression and may be related to the various mechanisms of antigen production and antigen processing pathways. Specifically, the relative presence of one isotype of an anti-MAA antibody over another is suggestive of a very unique immune response. It is enticing to speculate that at low levels of MAA-adduction, MAA-adducted proteins are rapidly cleared via local scavenger receptors with IgM stimulation and no antibody class switching. In contrast, with chronic and repeated tissue injury, there would be an expected increase in MAA-adduction and a shift in the pathways of scavenger receptor clearance that possibly result in B-cell activation and Ig class switching. A pathologic class switching to IgG would be expected to further increase local and systemic inflammation and result in MAA-modified LDL immune complexes that were associated with future cardiovascular events as presented by Lopes-Virella et al. [32].

Such changes in antigen processing, may also reflect mechanisms by which an isotype class switching results in various anti-MAA IgM, IgG and/or IgA such as: 1) The concentration of the MAA-modified antigen; 2) The density of MAA-adduction; 3) The specific amino acid(s) which is MAA-adducted, and 4) The immune tissue wherein immune sensitization occurs. For example, release of MAA-adducted protein into the plasma with immune complex formation and splenic immune sensitization would be expectedly different than when MAA-adducted proteins are released into interstitial spaces with immune sensitization in a lymphatic tissue bed. As well, the specific amino acid(s) of an individual protein which is MAA-modified may determine the extent of immune complex formation, Ig class switching, antibody recombination and affinity, and pathogenicity.

Levels of IgA anti-MAA have been recently reported to be associated with diabetes mellitus [30], and anti-oxidized LDL IgA has been reported with markers of glucose metabolism [31]. In our disease groups there is a significant proportion of diabetes present (Table 1). Despite less diabetes in our Non-Obstructive CAD patients as compared to AMI and Obstructive Multi-vessel CAD patients, the IgA anti-MAA relative levels were not significantly different between Non-Obstructive CAD and AMI. However, IgA levels are markedly different when compared to Obstructive Multi-vessel CAD (Figure 1C). This increase in IgA anti-MAA with diabetes supports the previously reported association of IgA with diabetes [30], [31], but does not fully address the marked increase in IgA anti-MAA in our Obstructive Multi-Vessel CAD patients as compared to AMI patients.

Another issue that needs further investigation is whether the nature of the immune response to MAA is dependent upon the specific cardiovascular protein that is MAA-modified. We hypothesize that there will be a robust immune response to the adducted cardiovascular protein as presented in Figure 4. Antibody levels for such protein(s) are expected to be much more specific for each cardiovascular disease compared to the relative levels of anti-MAA (presented in this report) which reflect a more systemic measure of inflammation, beta-oxidation of lipids and lipid peroxidation. Thus, even though anti-MAA antibodies are associated with cardiovascular disease, the identification of the specific cardiovascular proteins which are MAA-modified will be important in refining the phenotype and specificity of the acquired immune response to the MAA structure and/or the cardiovascular protein which is MAA-modified.

Figure 3 illustrates the decrease in anti-MAA relative levels twenty-four hour’s post-AMI without a decrease in the total serum Ig concentration. This IgM and IgG anti-MAA antibody level decrease (Figure 3A) after AMI is thought to be due to the release of MAA-modified proteins, complement activation [50], MAA-adduct immune complex formation [32] and clearance, as a result of cardiac tissue ischemia, revascularization and reperfusion. This anti-MAA serum concentration decrease is consistent with previously reported studies by Segev et al. which evaluated the changes in serum antibody concentrations secondary to a cardiovascular event and an increase in serum antigens [51]. Of note, IgA antibodies did not decrease at 24 hours post-AMI, and this is consistent with the inability of serum IgA to activate complement, form MAA-adduct immune complexes, and be cleared from the circulation. In this case, the inflammatory milieu would be expectedly different than if IgM and/or IgG predominated. Thus, the stability in IgA antibody levels with an AMI as compared to IgM and IgG anti-MAA antibody levels is of great interest. It will be important to understand in greater detail the stability of IgA over time, with or without changes in IgM and IgG, affords a reliable measure of disease activity and/or risk of a cardiovascular event.

This data shows the importance in evaluating MAA-specific isotype levels in a larger cohort of patients who are at risk for cardiovascular events to determine the predictive value of this bioassay. MAA-modified proteins may serve as mediators of cardiovascular tissue dysfunction, inflammation and as antigens in the innate and acquired immunity of vascular inflammation. The impact of MAA-adduction on tissue inflammation and the clinical progression of atherosclerotic disease warrant further mechanistic studies of MAA-adduction and cardiovascular risk.

## Conclusions

Data from this pilot cohort of patients shows a significant increase in the relative levels of circulating anti-MAA antibodies and the presence of MAA adducts in the atheromas of patients with early and advanced atherosclerosis which is different than anti-MDA-LDL and anti-MAA-LDL antibody levels. Specifically, 1) CAD patients have elevated levels of IgG and IgA anti-MAA antibodies compared to control patients; 2) AMI patients have a significantly increased level of circulating IgM and IgG anti-MAA antibodies compared to Non-Obstructive CAD or CABG patients; 3) CABG patients have significantly increased levels of circulating IgA anti-MAA antibodies compared to Non-Obstructive CAD or AMI patients; 4) IgA anti-MAA antibody levels are stable 24 hours after an AMI as compared to IgM and IgG anti-MAA antibodies; 5) MAA-modified proteins are present within the tissue of the culprit AMI atheromatous lesion; 6) MAA-adduction and humoral immune sensitization to the MAA structure, may be a mechanism for sensitization to MAA-adducted cardiovascular proteins, to the hapten-carrier structure of the MAA-adducted cardiovascular protein, and to other co-adducts, and 7) MAA-adduction is present in non-cardiovascular tissues and may represent a global paradigm of tissue injury, inflammation and repair that if in excess (due to increases production or decreased clearance) over time is associated with tissue specific disease and/or accelerated progression of disease.

Anti-MAA antibody isotypes and MAA-modified proteins may serve as mechanistic biomarkers of atherosclerotic disease, may allow earlier detection and differentiation of CAD progression (i.e. stable vs. unstable CAD), may allow earlier treatment of CAD, and may allow for the assessment of a future cardiovascular event. A larger cohort of patients who are followed in a prospective fashion is needed to verify these associations.

### Study Limitations

This pilot study and hypothesis generating manuscript is limited in the cohort sample size and places an emphasis on the validation of these findings in a larger cohort of patients which are prospectively followed for cardiovascular events.

## References

[1] Libby P, Ridker PM, Hansson GK (2009) Inflammation in atherosclerosis: from pathophysiology to practice. Journal of the American College of Cardiology 54: 2129–2138. 19942084 10.1016/j.jacc.2009.09.009 PMC2834169

[2] Ross R (1999) Atherosclerosis–an inflammatory disease. The New England journal of medicine 340: 115–126. 9887164 10.1056/NEJM199901143400207

[3] Anderson DR, Poterucha JT, Mikuls TR, Duryee MJ, Garvin RP, et al (2013) IL-6 and its receptors in coronary artery disease and acute myocardial infarction. Cytokine 62: 395–400. 23582716 10.1016/j.cyto.2013.03.020

[4] Ridker PM, Danielson E, Fonseca FA, Genest J, Gotto AM Jr, et al (2008) Rosuvastatin to prevent vascular events in men and women with elevated C-reactive protein. The New England journal of medicine 359: 2195–2207. 18997196 10.1056/NEJMoa0807646

[5] Duryee MJ, Klassen LW, Schaffert CS, Tuma DJ, Hunter CD, et al (2010) Malondialdehyde-acetaldehyde adduct is the dominant epitope after MDA modification of proteins in atherosclerosis. Free radical biology & medicine 49: 1480–1486. 20696236 10.1016/j.freeradbiomed.2010.08.001 PMC2952714

[6] Fu S, Davies MJ, Stocker R, Dean RT (1998) Evidence for roles of radicals in protein oxidation in advanced human atherosclerotic plaque. The Biochemical journal 333 (Pt 3): 519–525. 10.1042/bj3330519 PMC1219612 9677308

[7] Heinecke JW (1998) Oxidants and antioxidants in the pathogenesis of atherosclerosis: implications for the oxidized low density lipoprotein hypothesis. Atherosclerosis 141: 1–15. 9863534 10.1016/s0021-9150(98)00173-7

[8] Hill GE, Miller JA, Baxter BT, Klassen LW, Duryee MJ, et al (1998) Association of malondialdehyde-acetaldehyde (MAA) adducted proteins with atherosclerotic-induced vascular inflammatory injury. Atherosclerosis 141: 107–116. 9863543 10.1016/s0021-9150(98)00153-1

[9] Uchida K (2006) Lipofuscin-like fluorophores originated from malondialdehyde. Free radical research 40: 1335–1338. 17090422 10.1080/10715760600902302

[10] Holvoet P (1998) Oxidative modification of low-density lipoproteins in atherothrombosis. Acta cardiologica 53: 253–260. 9922802

[11] Palinski W, Horkko S, Miller E, Steinbrecher UP, Powell HC, et al (1996) Cloning of monoclonal autoantibodies to epitopes of oxidized lipoproteins from apolipoprotein E-deficient mice. Demonstration of epitopes of oxidized low density lipoprotein in human plasma. The Journal of clinical investigation 98: 800–814. 8698873 10.1172/JCI118853 PMC507491

[12] Palinski W, Rosenfeld ME, Yla-Herttuala S, Gurtner GC, Socher SS, et al (1989) Low density lipoprotein undergoes oxidative modification in vivo. Proceedings of the National Academy of Sciences of the United States of America 86: 1372–1376. 2465552 10.1073/pnas.86.4.1372 PMC286692

[13] Yla-Herttuala S, Palinski W, Rosenfeld ME, Parthasarathy S, Carew TE, et al (1989) Evidence for the presence of oxidatively modified low density lipoprotein in atherosclerotic lesions of rabbit and man. The Journal of clinical investigation 84: 1086–1095. 2794046 10.1172/JCI114271 PMC329764

[14] Herrmann J, Soares SM, Lerman LO, Lerman A (2008) Potential role of the ubiquitin-proteasome system in atherosclerosis: aspects of a protein quality disease. Journal of the American College of Cardiology 51: 2003–2010. 18498952 10.1016/j.jacc.2008.02.047

[15] Ketelhuth DF, Hansson GK (2011) Cellular immunity, low-density lipoprotein and atherosclerosis: break of tolerance in the artery wall. Thromb Haemost 106: 779–786. 21979058 10.1160/TH11-05-0321

[16] Liu ZD, Wang L, Lu FH, Pan H, Zhao YX, et al (2012) Increased Th17 cell frequency concomitant with decreased Foxp3+ Treg cell frequency in the peripheral circulation of patients with carotid artery plaques. Inflamm Res 61: 1155–1165. 22728962 10.1007/s00011-012-0510-2

[17] Butcher MJ, Gjurich BN, Phillips T, Galkina EV (2012) The IL-17A/IL-17RA axis plays a proatherogenic role via the regulation of aortic myeloid cell recruitment. Circ Res 110: 675–687. 22302786 10.1161/CIRCRESAHA.111.261784 PMC3337709

[18] Danzaki K, Matsui Y, Ikesue M, Ohta D, Ito K, et al (2012) Interleukin-17A deficiency accelerates unstable atherosclerotic plaque formation in apolipoprotein E-deficient mice. Arterioscler Thromb Vasc Biol 32: 273–280. 22116098 10.1161/ATVBAHA.111.229997

[19] Gao Q, Jiang Y, Ma T, Zhu F, Gao F, et al (2010) A critical function of Th17 proinflammatory cells in the development of atherosclerotic plaque in mice. J Immunol 185: 5820–5827. 20952673 10.4049/jimmunol.1000116 PMC12230985

[20] Lopes-Virella MF, Virella G (2010) Clinical significance of the humoral immune response to modified LDL. Clinical immunology 134: 55–65. 19427818 10.1016/j.clim.2009.04.001 PMC2808452

[21] Ravandi A, Boekholdt SM, Mallat Z, Talmud PJ, Kastelein JJ, et al (2011) Relationship of IgG and IgM autoantibodies and immune complexes to oxidized LDL with markers of oxidation and inflammation and cardiovascular events: results from the EPIC-Norfolk Study. Journal of lipid research 52: 1829–1836. 21821825 10.1194/jlr.M015776 PMC3173004

[22] Tsimikas S, Brilakis ES, Lennon RJ, Miller ER, Witztum JL, et al (2007) Relationship of IgG and IgM autoantibodies to oxidized low density lipoprotein with coronary artery disease and cardiovascular events. Journal of lipid research 48: 425–433. 17093289 10.1194/jlr.M600361-JLR200

[23] Tsimikas S, Bergmark C, Beyer RW, Patel R, Pattison J, et al (2003) Temporal increases in plasma markers of oxidized low-density lipoprotein strongly reflect the presence of acute coronary syndromes. J Am Coll Cardiol 41: 360–370. 12575961 10.1016/s0735-1097(02)02769-9

[24] Tsimikas S, Witztum JL, Miller ER, Sasiela WJ, Szarek M, et al (2004) High-dose atorvastatin reduces total plasma levels of oxidized phospholipids and immune complexes present on apolipoprotein B-100 in patients with acute coronary syndromes in the MIRACL trial. Circulation 110: 1406–1412. 15353498 10.1161/01.CIR.0000141728.23033.B5

[25] Tuma DJ, Thiele GM, Xu D, Klassen LW, Sorrell MF (1996) Acetaldehyde and malondialdehyde react together to generate distinct protein adducts in the liver during long-term ethanol administration. Hepatology 23: 872–880. 8666344 10.1002/hep.510230431

[26] Thiele GM, Tuma DJ, Willis MS, Miller JA, McDonald TL, et al (1998) Soluble proteins modified with acetaldehyde and malondialdehyde are immunogenic in the absence of adjuvant. Alcoholism, clinical and experimental research 22: 1731–1739. 9835288

[27] Willis MS, Klassen LW, Tuma DJ, Sorrell MF, Thiele GM (2002) Adduction of soluble proteins with malondialdehyde-acetaldehyde (MAA) induces antibody production and enhances T-cell proliferation. Alcohol Clin Exp Res 26: 94–106. 11821659

[28] Tuma DJ, Newman MR, Donohue TM Jr, Sorrell MF (1987) Covalent binding of acetaldehyde to proteins: participation of lysine residues. Alcohol Clin Exp Res 11: 579–584. 3124658 10.1111/j.1530-0277.1987.tb00178.x

[29] Willis MS, Klassen LW, Carlson DL, Brouse CF, Thiele GM (2004) Malondialdehyde-acetaldehyde haptenated protein binds macrophage scavenger receptor(s) and induces lysosomal damage. International immunopharmacology 4: 885–899. 15182728 10.1016/j.intimp.2004.04.004

[30] Vehkala L, Ukkola O, Kesaniemi YA, Kahonen M, Nieminen MS, et al (2013) Plasma IgA antibody levels to malondialdehyde acetaldehyde-adducts are associated with inflammatory mediators, obesity and type 2 diabetes. Ann Med 45: 501–510. 24131174 10.3109/07853890.2013.841322

[31] Sampi M, Veneskoski M, Ukkola O, Kesaniemi YA, Horkko S (2010) High plasma immunoglobulin (Ig) A and low IgG antibody titers to oxidized low-density lipoprotein are associated with markers of glucose metabolism. J Clin Endocrinol Metab 95: 2467–2475. 20332251 10.1210/jc.2009-1858

[32] Lopes-Virella MF, Hunt KJ, Baker NL, Virella G, Moritz T, et al (2012) The levels of MDA-LDL in circulating immune complexes predict myocardial infarction in the VADT study. Atherosclerosis 224: 526–531. 22963984 10.1016/j.atherosclerosis.2012.08.006 PMC4240617

[33] Furie KL, Goldstein LB, Albers GW, Khatri P, Neyens R, et al (2012) Oral antithrombotic agents for the prevention of stroke in nonvalvular atrial fibrillation: a science advisory for healthcare professionals from the American Heart Association/American Stroke Association. Stroke 43: 3442–3453. 22858728 10.1161/STR.0b013e318266722a

[34] Xu D, Thiele GM, Kearley ML, Haugen MD, Klassen LW, et al (1997) Epitope characterization of malondialdehyde-acetaldehyde adducts using an enzyme-linked immunosorbent assay. Chemical research in toxicology 10: 978–986. 9305579 10.1021/tx970069t

[35] Willerson JT, Ridker PM (2004) Inflammation as a cardiovascular risk factor. Circulation 109: II2–10. 15173056 10.1161/01.CIR.0000129535.04194.38

[36] Ridker PM, Rifai N, Rose L, Buring JE, Cook NR (2002) Comparison of C-reactive protein and low-density lipoprotein cholesterol levels in the prediction of first cardiovascular events. N Engl J Med 347: 1557–1565. 12432042 10.1056/NEJMoa021993

[37] Rolla R, Vay D, Mottaran E, Parodi M, Traverso N, et al (2000) Detection of circulating antibodies against malondialdehyde-acetaldehyde adducts in patients with alcohol-induced liver disease. Hepatology 31: 878–884. 10733543 10.1053/he.2000.5373

[38] Palinski W, Yla-Herttuala S, Rosenfeld ME, Butler SW, Socher SA, et al (1990) Antisera and monoclonal antibodies specific for epitopes generated during oxidative modification of low density lipoprotein. Arteriosclerosis 10: 325–335. 1693068 10.1161/01.atv.10.3.325

[39] Horkko S, Binder CJ, Shaw PX, Chang MK, Silverman G, et al (2000) Immunological responses to oxidized LDL. Free radical biology & medicine 28: 1771–1779. 10946219 10.1016/s0891-5849(00)00333-6

[40] Tsimikas S, Willeit P, Willeit J, Santer P, Mayr M, et al (2012) Oxidation-specific biomarkers, prospective 15-year cardiovascular and stroke outcomes, and net reclassification of cardiovascular events. J Am Coll Cardiol 60: 2218–2229. 23122790 10.1016/j.jacc.2012.08.979

[41] Mikuls TR, Holers VM, Parrish L, Kuhn KA, Conn DL, et al (2006) Anti-cyclic citrullinated peptide antibody and rheumatoid factor isotypes in African Americans with early rheumatoid arthritis. Arthritis Rheum 54: 3057–3059. 16948136 10.1002/art.22200

[42] Milioti N, Bermudez-Fajardo A, Penichet ML, Oviedo-Orta E (2008) Antigen-induced immunomodulation in the pathogenesis of atherosclerosis. Clin Dev Immunol 2008: 723539. 18551190 10.1155/2008/723539 PMC2423423

[43] Aikawa M, Libby P (2004) The vulnerable atherosclerotic plaque: pathogenesis and therapeutic approach. Cardiovasc Pathol 13: 125–138. 15081469 10.1016/S1054-8807(04)00004-3

[44] Willis MS, Klassen LW, Tuma DJ, Sorrell MF, Thiele GM (2002) In vitro exposure to malondialdehyde-acetaldehyde adducted protein inhibits cell proliferation and viability. Alcohol Clin Exp Res 26: 158–164. 11964554

[45] Willis MS, Thiele GM, Tuma DJ, Klassen LW (2003) T cell proliferative responses to malondialdehyde-acetaldehyde haptenated protein are scavenger receptor mediated. Int Immunopharmacol 3: 1381–1399. 12946435 10.1016/S1567-5769(03)00136-X

[46] Choudhary C, Kumar C, Gnad F, Nielsen ML, Rehman M, et al (2009) Lysine acetylation targets protein complexes and co-regulates major cellular functions. Science 325: 834–840. 19608861 10.1126/science.1175371

[47] Sokolove J, Brennan MJ, Sharpe O, Lahey LJ, Kao AH, et al (2013) Brief report: citrullination within the atherosclerotic plaque: a potential target for the anti-citrullinated protein antibody response in rheumatoid arthritis. Arthritis Rheum 65: 1719–1724. 23553485 10.1002/art.37961 PMC3731137

[48] Kummu O, Turunen SP, Prus P, Lehtimaki J, Veneskoski M, et al (2014) Human monoclonal Fab and human plasma antibodies to carbamyl-epitopes cross-react with malondialdehyde-adducts. Immunology 141: 416–430. 24168430 10.1111/imm.12204 PMC3930379

[49] Newby AC (2008) Metalloproteinase expression in monocytes and macrophages and its relationship to atherosclerotic plaque instability. Arterioscler Thromb Vasc Biol 28: 2108–2114. 18772495 10.1161/ATVBAHA.108.173898

[50] Veneskoski M, Turunen SP, Kummu O, Nissinen A, Rannikko S, et al (2011) Specific recognition of malondialdehyde and malondialdehyde acetaldehyde adducts on oxidized LDL and apoptotic cells by complement anaphylatoxin C3a. Free Radic Biol Med 51: 834–843. 21683785 10.1016/j.freeradbiomed.2011.05.029

[51] Segev A, Strauss BH, Witztum JL, Lau HK, Tsimikas S (2005) Relationship of a comprehensive panel of plasma oxidized low-density lipoprotein markers to angiographic restenosis in patients undergoing percutaneous coronary intervention for stable angina. Am Heart J 150: 1007–1014. 16290986 10.1016/j.ahj.2004.12.008

